# Metabolic and molecular insights into an essential role of nicotinamide phosphoribosyltransferase

**DOI:** 10.1038/cddis.2017.132

**Published:** 2017-03-23

**Authors:** Li Q Zhang, Leon Van Haandel, Min Xiong, Peixin Huang, Daniel P Heruth, Charlie Bi, Roger Gaedigk, Xun Jiang, Ding-You Li, Gerald Wyckoff, Dmitry N Grigoryev, Li Gao, Linheng Li, Min Wu, J Steven Leeder, Shui Qing Ye

**Affiliations:** 1 Division of Experimental and Translational Genetics, Department of Pediatrics, The Children's Mercy Hospital, University of Missouri – Kansas City School of Medicine, Kansas City, MO, USA; 2 Division of Clinical Pharmacology and Therapeutic Innovation,The Children's Mercy Hospital, Kansas City, MO, USA; 3 Department of Pediatrics, Tangdu Hospital, Fourth Military Medical University, Xian, China; 4 Division of Gastroenterology, Department of Pediatrics, The Children's Mercy Hospital, Kansas City, MO, USA; 5 Division of Molecular Biology and Biochemistry, University of Missouri School of Biological Sciences, Kansas City, MO, USA; 6 Laboratory of Genitourinary Cancer Pathogenesis, National Cancer Institute, Bethesda, MD, USA; 7 Division of Allergy and Clinical Immunology, Johns Hopkins University School of Medicine, Baltimore, MD, USA; 8 Stowers Institute for Medical Research, Kansas City, MO, USA; 9 Department of Biomedical Sciences, University of North Dakota, Grand Forks, ND, USA; 10 Department of Biomedical and Health Informatics, University of Missouri – Kansas City School of Medicine, Kansas City, MO, USA

## Abstract

Nicotinamide phosphoribosyltransferase (NAMPT) is a pleiotropic protein implicated in the pathogenesis of acute respiratory distress syndrome, aging, cancer, coronary heart diseases, diabetes, nonalcoholic fatty liver disease, obesity, rheumatoid arthritis, and sepsis. However, the underlying molecular mechanisms of NAMPT in these physiological and pathological processes are not fully understood. Here, we provide experimental evidence that a *Nampt* gene homozygous knockout (*Nampt*^−/−^) resulted in lethality at an early stage of mouse embryonic development and death within 5–10 days in adult mice accompanied by a 25.24±2.22% body weight loss, after the tamoxifen induction of Nampt^F/F^ × Cre mice. These results substantiate that *Nampt* is an essential gene for life. In *Nampt*^−/−^ mice *versus* *Nampt*^+/+^ mice, biochemical assays indicated that liver and intestinal tissue NAD levels were decreased significantly; histological examination showed that mouse intestinal villi were atrophic and disrupted, and visceral fat was depleted; mass spectrometry detected unusual higher serum polyunsaturated fatty acid containing triglycerides. RNA-seq analyses of both mouse and human pediatric liver transcriptomes have convergently revealed that NAMPT is involved in key basic cellular functions such as transcription, translation, cell signaling, and fundamental metabolism. Notably, the expression of all eight enzymes in the tricarboxylic acid cycle were decreased significantly in the *Nampt*^−/−^ mice. These findings prompt us to posit that adult *Nampt*^−/−^ mouse lethality is a result of a short supply of ATP from compromised intestinal absorption of nutrients from digested food, which leads to the exhaustion of body fat stores.

Nicotinamide phosphoribosyltransferase (NAMPT) was initially named pre-B-cell colony-enhancing factor (PBEF)^1^ as it synergized the pre-B-cell colony-formation activity of stem cell factor and interleukin 7. NAMPT catalyzes the condensation of nicotinamide with 5-phosphoribosyl-1-pyrophosphate to yield nicotinamide mononucleotide, an intermediate in the biosynthesis of NAD.^2^ In 2005, Fukuhara *et al.*^3^ reported Nampt as a Visfatin, which was enriched in visceral fat with insulin-mimetic function. The list of NAMPT's physiological roles is continuously growing. The dysregulation of *NAMPT* gene has been implicated in several human diseases.^4^ However, the underlying molecular mechanisms are not fully understood.

Loss-of-function experiments reveal the functions of a gene. The most dramatic phenotype is lethality, defining a gene as essential.^5^ Recently, three groups reported that there are about 2000 core genes essential for life in several types of human cells.^6, 7, 8^ They also found that the essentiality of some genes is context-dependent and affects viability in a cell-type-specific manner.^9^ It is more logical to establish any essential gene in an animal model, by exploring the terminal phenotypes and the various molecular mechanisms underlying lethality. The mouse has long been regarded as an ideal model system for this purpose. Conservation between humans and mice is underscored by strong one-to-one orthologous relationships between genes of the two species.^10^ We have previously reported that the *Nampt* gene DNA coding sequence between mouse and human is 96% identical.^11^ Hence, the homozygous *Nampt* gene knockout in mice would offer an attractive model to decipher fully the *Nampt* gene's pleiotropic roles, by probing molecular mechanisms underlying lethality in adult mice.

Here, we provide experimental evidence that the *Nampt* gene homozygous knockout (*Nampt*^−/−^) resulted in mouse embryonic lethality at an early stage. We also established *Nampt* gene conditional knockout mice and found that induced *Nampt*^−/−^ adult mice died within 5–10 days, accompanied by a 25.24±2.22% body weight loss. These results substantiate that *Nampt* is an essential gene *in vivo*. RNA-seq analyses of both mouse and human pediatric liver transcriptomes revealed that *Nampt* is involved in key basic cellular functions such as transcription, translation, cell signaling, and fundamental metabolism. Particularly, expression of all eight enzymes in the tricarboxylic acid cycle(TAC) in *Nampt*^−/−^ adult mice were decreased significantly, which suggests that depletion of cellular ATP energy sources may be the main culprit of adult Nampt^−/−^ mouse death.

## Results

### Homozygous *Nampt* gene knockout mice are embryonically lethal

**Figure 1:**
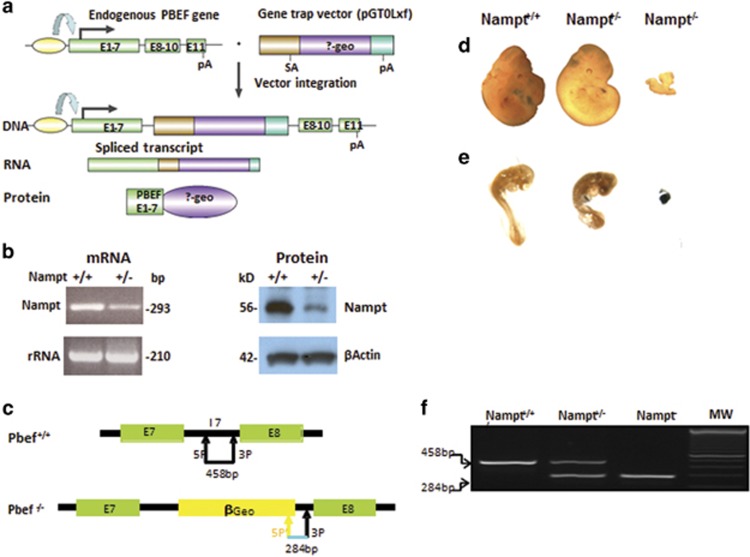
Homozygous *Nampt* gene knockout mice are embryonically lethal. (**a**) The schematics of gene-trap strategy to create *Nampt* knockout mice. Gene-trap cassette were inserted in *Nampt* gene intron-7 and the transcription of the ‘trapped' *Nampt* gene results in a truncated mRNA that will translate into a *Nampt* exon 1–7 and *β*-geo fusion protein. (**b**) Phenotypic verification of *Nampt* gene-trapped embryonic stem (ES) cell line. Phenotypic verifications of an ES cell line (RRR084) with *Nampt* gene interruption (*Nampt*^+/−)^ were carried out by RT-PCR for *Nampt* mRNA and western blotting for *Nampt* protein expression. *Nampt*^+/+^ represents wild-type ES cell line without a gene-trap vector insertion. (**c**) Genotyping strategy. Mouse yolk sac or tail DNA was used for genotyping by PCR. The locations of *Nampt* gene intron-7 and *β*-Geo specific primers used for genotyping and predicted size of the PCR products are shown. (**d**) Representative mouse embryos at 11.5 d.p.c. Timed pregnancies and embryo collection were carried out as described in the 'Materials and Methods' section. Mouse embryo images from representative *Nampt*^+/+^ , *Nampt*^+/−^, and *Nampt*^−/−^ mice at 11.5 d.p.c. are displayed. (**e**) Representative mouse embryos at 8.5 d.p.c. Timed pregnancies and embryo collection were carried out as described in the 'Materials and Methods' section. Mouse embryo images from representative *Nampt*^+/+^, *Nampt*^+/−^, and *Nampt*^−/−^ mice at 8.5 d.p.c. after *β*-Gal staining are displayed. (**f**) Representative genotyping images. Mouse genotyping was done using PCR amplification of genomic DNA isolated from mouse embryonic tissue or yolk sac with either primer pairs 5P and 3P or 5P' and 3P as depicted in **c**. The PCR products were subjected to 2% agarose electrophoresis and stained by ethidium bromide

To study the biological functions of the *Nampt* gene, we established the *Nampt* gene-trapped mouse model (Figure 1a). To confirm the mutant ES cell line, RT-PCR results showed that the cell line only expresses about 50% *Nampt* mRNA of the *Nampt*^+/+^ wild-type control, and western blotting demonstrated that cellular Nampt protein in the *Nampt*^+/−^ ES cell line is decreased significantly (Figure 1b). The validated ES cell lines were injected into blastocysts of pseudopregnant female C57BL/6N mice at the Johns Hopkins University transgenic mouse core facility to generate the *Nampt*^+/−^ knockdown mouse model. To facilitate mouse genotyping needs, we sequenced the whole region of *Nampt* gene intron-7, including the inserted gene-trap cassette (Supplementary Figure 1), and designed three PCR primers (two forward and one reverse) to detect *Nampt* wild-type (*Nampt*^+/+^), *Nampt* heterozygous (*Nampt*^+/−^), and *Nampt* knockout (*Nampt*^−/−^) mice as indicated in Figure 1c. Representative genotyping images of *Nampt*^+/+^, *Nampt*^+/−^, and Nampt^−/−^ mice are presented in Figure 1f. To generate *Nampt*^−/−^ knockout mice, *Nampt*^+/−^ male and female mice were bred. At embryonic day 11.5, *Nampt*^+/+^ and *Nampt*^+/−^ embryos both have normal development with 40–45 pairs of somites, and normal embryo size. *Nampt*^−/−^ embryos are much smaller in size, and display unclear abnormal embryo structure (Figure 1d). To establish the Nampt^−/−^ embryonic lethality, we performed a *β*-gal staining of mouse embryos at 8.5 d.p.c. stage, as the inserted gene-trap vector *β*-Geo can express *β*-gal. A representative normal size *Nampt*^+/−^ embryo has a diffused brown color staining as in the *Nampt*^+/+^ embryo, while an undeveloped *Nampt*^−/−^ embryo shows strong dark blue staining (Figure 1e). These results corroborate that the *Nampt*^−/−^ mice are lethal at an early embryonic stage.

### *Nampt* is an essential gene for adult mouse survival

**Figure 2:**
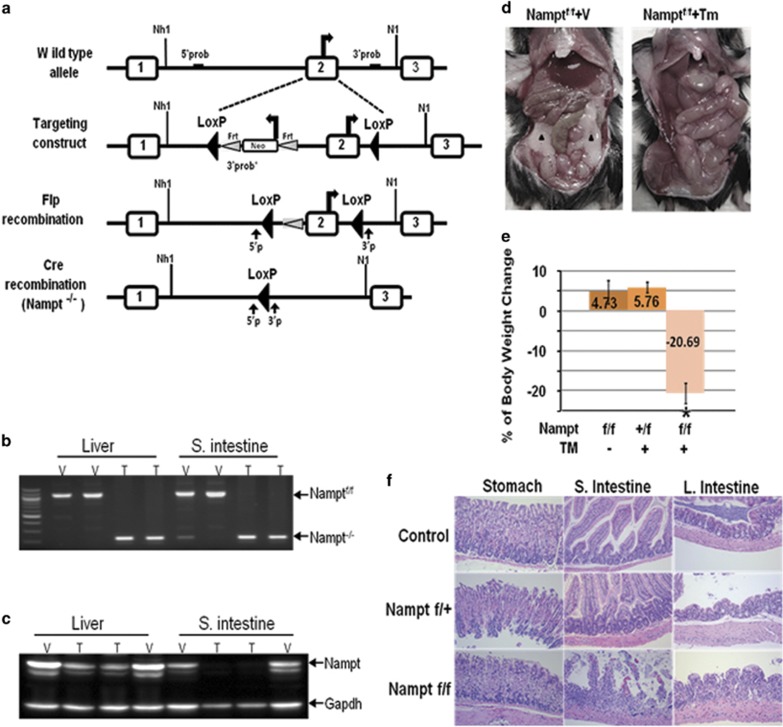
*Nampt* is an essential gene for adult mouse survival. (**a**) Gene targeting strategy for production of mice for conditional inactivation of *Nampt* gene. Part of intron-1 of the *Nampt* gene was used as 5′ arm, part of intron-2 as 3′ arm, exon 2 floxed, Neo and TK used as positive and negative selection marker, respectively. In the process of homologous recombination, targeting construct replaced the exon 2 of *Nampt* gene. Neo was selectively removed by Flipase recombinase. Cre-Lox recombination leads to *Nampt* second exon excision. 5′P and 3′P represent mice genotyping PCR primers and the black arrows indicate the location of the primers. (**b**) Genotyping of *Nampt*^f/f^ and *Nampt*^−/−^ mice. Representative images of genotyping of *Nampt*^f^/^f^ and *Nampt*^−/−^ mice by PCR amplification of either liver or small intestine tissue DNAs are shown. T, tamoxifen; V, vehicle. (**c**) *Nampt* protein expression in *Nampt*^f/f^ and *Nampt*^−/−^ mice. Western blotting detecting the *Nampt* protein expression in mouse liver and small intestine tissues are shown. Gapdh was included as a loading control. (**d**) Gross morphology of TMX-treated or control mouse abdominal visceral tissues. After TMX (0.04 mg/kg/day, ip) injection for 4 days, mice body weight was closely monitored. All the *Ubc*^*CreERT2*^*; Nampt*^flox/flox^ mice treated with TMX will loss 20% the body weight within 5 days. Just before mouse morbid, mouse abdomen were dissected as shown. Black arrow head indicate the visceral adipose tissues in a vehicle control mouse. (**e**) Statistical analysis of mouse body weight changes. There are two groups of control mice, *Nampt*^f/f^ treated with vehicle and *Nampt*^+/f^ treated with TMX, both are compared with *Nampt*^f/f^ mice treated with TMX; *N*=10 for each group. *P<0.01 *Nampt*^−/−^*versus* either Nampt^f/f^ or *Nampt*^+/f^ group. (**f**) Histology of gastrointestinal track by H&E staining. Amplification, × 100; TMX, tamoxifen; Veh, vehicle

As *Nampt*^−/−^ mice are embryonically lethal, we cannot use *Nampt*^−/−^ mice for a complete ‘loss of function' study and hence we generated a *Nampt* conditional knockout mouse line (*Namp*^F/F^). The *Nampt* gene spans over 36.9 kb and contains 11 exons and 10 introns, thus preventing the convenient floxing of its entire gene. We decided to introduce *loxP* sites into introns flanking exon 2. A *Neo* selection cassette flanked by *FRT* sites was inserted adjacent of the *loxP* site within intron-1 as well (Figure 2a). The *Nampt*^flox(Neo)^ targeting vector was introduced into mouse embryonic stem cells (mES), which were then injected into C57BL/6 host blastocysts to generate chimeric mice (Supplementary Figure 2). *Nampt*^flox(Neo)/+^ heterozygous mice were crossed with the *ACTB*::*FLPe* deleter mouse line to remove the *PGK-Neo* cassette, which has been reported to interfere with gene expression (Figure 2a). *Nampt*^flox/+^ mice were backcrossed with C57BL/6J wild-type mice for 10 generations before being considered as a pure C57BL/6 background, and subsequently intercrossed to obtain homozygous *Nampt*^flox/flox^ mice. All the genotypes were confirmed by PCR analysis (Figure 2b) and tissue expression levels of Nampt protein were verified by western blotting (Figure 2c). *Nampt*^flox/flox^ mice were born at a normal Mendelian ratio and were viable without displaying any obvious defect. To conditionally induce cre-loxP recombination, *Nampt*^flox/flox^ mice were mated to *Ubc*^*CreERT2*^ mice. After 4-day intraperitoneal administration of tamoxifen (TMX), *Nampt*^flox/flox^ mice (*n*=10) displayed rapid weight loss. These mice became dehydrated, less active on day 3 after injection and reached >20% weight loss within 5–10 days before dying (Figure 2e). Dissection of dying *Nampt*^−/−^ mice showed fat tissue depletion and intestinal abnormality in TMX-treated *Ubc*^*CreERT2*^*: Nampt*^*flox*/flox^ mice (Figure 2d). The Nampt protein expression levels were dramatically reduced in TMX-treated *Ubc*^*CreERT2*^*: Nampt*^*flox*/flox^ mice after day 5(Figure 2c). Histological examination revealed that *Nampt* depletion induced severe intestinal villus atrophy and the disruption of normal architecture (Figure 2f) compared with the wild-type control mice.

### Abnormal serum lipid profile in *Nampt*^−/−^ mice

**Figure 3:**
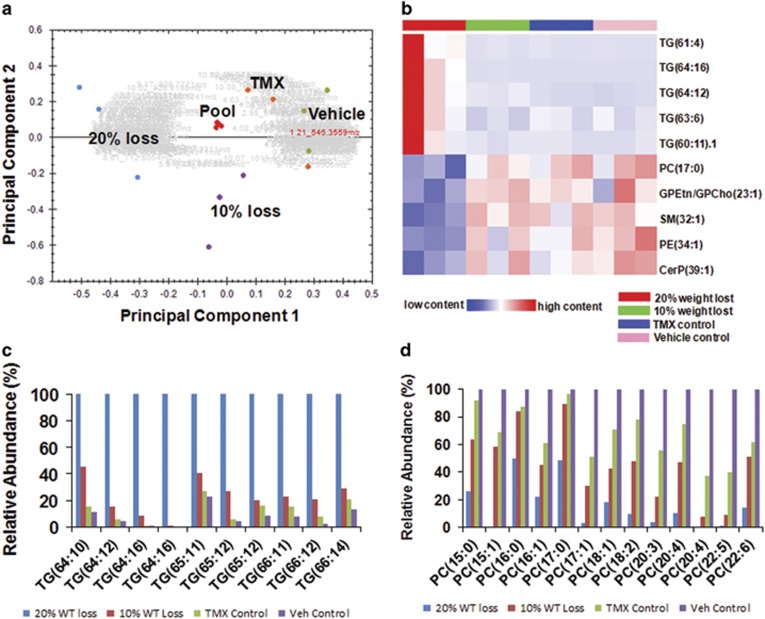
Serum lipid profiles in *Nampt*^+/+^ and *Nampt*^−/−^ mice. (**a**) PCA analysis of mouse serum lipid profiles. PCA score plot is derived from MS spectrum of serum samples from vehicle control (vehicle •), tamoxifen control (TMX •), 10% weight loss (10% loss •), 20% weight loss (20% loss •), and pooled samples (Pool •). *N*=3 per group. (**b**) Heatmap of differentially expressed serum lipids in mice. Heatmap of the top five upregulated and the top five downregulated differentially expressed lipids between the *Nampt* knockdown (20% weight loss, 10% weight loss) and control (tamoxifen and vehicle) groups is presented. (**c**) Relative abundance of mouse serum triacylglycerides (TG). Relative abundance of TG with increased number side chain carbons (⩾64) and double bonds between the four groups is represented. (**d**) Relative abundance of mouse serum phosphatidylcholines (PC). Relative abundance of PC with 15 to 22 side chain carbons between the four groups is represented

To characterize the rapid catabolism of visceral fat in *Nampt*^−/−^ mice, we analyzed the serum lipid content in these mice (Supplementary Table 1) by UHPLC-qTOF MS method. Principal component analysis revealed that each experimental group clustered as a unique entity (Figure 3a). One hundred forty-one lipids were significantly and differentially expressed among the four experimental groups (Supplementary Table 1 and Supplementary Figure 3). A heatmap of the top 10 differentially presented lipids is displayed in Figure 3b. High molecular weight Triacylglyceride (TG) with long polyunsaturated carbon side chains (~4 or more double bonds) were more prevalent in the 10 and 20% weight loss group compared with the control groups (Figure 3c,Supplementary Table 1 and Supplementary Figure 3). Conversely, TGs with a lower number of side chain carbons and fewer double bonds were less abundant in the control mice than in the mice with weight loss, especially the 20% weight loss group. In contrast to TG levels, phosphatidylcholine lipid (PC) levels were less abundant in the *Nampt* knockout mice with 10 and 20% weight loss relative to the tamoxifen and vehicle control mice (Figure 3d, Supplementary Table 1 and Supplementary Figure 3).

### Liver transcriptome profile in *Nampt*^−/−^ mice

**Figure 4:**
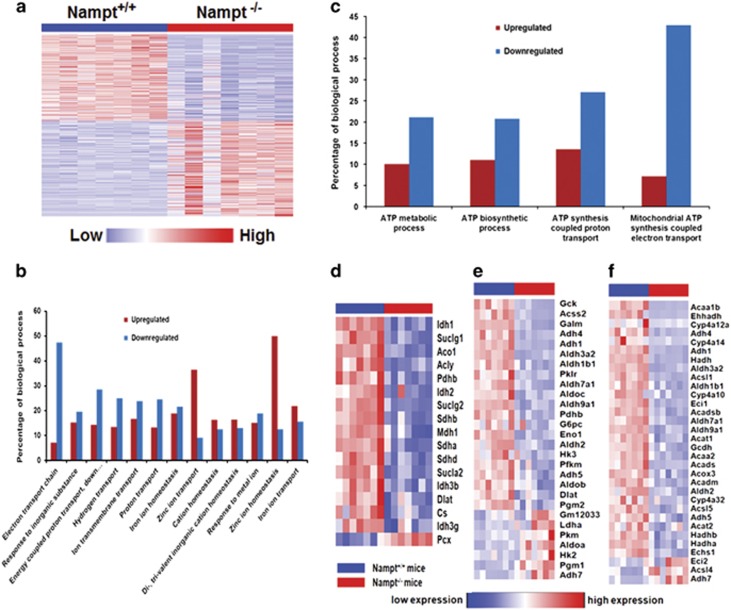
Liver transcription profiles in *Nampt*^−/−^ mice. (**a**) Heatmap of 4638 significantly differentially expressed liver genes in *Nampt*^−/−^ mice *versus Nampt*^+/+^ mice. Each column represents a mouse sample. Each row represents a gene. Red color indicates an increased expression. Blue color indicates a decreased expression. *N*=7 for each group. (**b**) Expression profile of significantly differentially expressed liver genes in 13-ion-associated transport biological processes of *Nampt*^−/−^ mice *versus Nampt*^+/+^ mice. Percentage value is derived from the number of significantly differentially expressed expression genes in each process divided by total genes involved in 13-ion-associated transport processes. (**c**) Expression profile of significantly differentially expressed liver genes in four ATP-associated metabolic processes of *Nampt*^−/−^ mice *versus Nampt*^+/+^ mice. Percentage value is derived from the number of significantly differentially expressed expression genes in each process divided by total genes involved in four ATP-associated metabolic processes. (**d**) Heatmap for significantly differentially expressed genes in citrate cycle in *Nampt*^−/−^ mice *versus Nampt*^+/+^ mice. (**e**) Heatmap for significantly differentially expressed genes in glycolysis and gluconeogenesis in *Nampt*^−/−^ mice *versus Nampt*^+/+^ mice. (**f**) Heatmap for significantly differentially expressed genes in fatty acid metabolism in *Nampt*^−/−^ mice *versus Nampt*^+/+^ mice. In all heat maps, red color indicates high expression, blue color indicates low expression. *N*=7 in each group. The expression of all genes in (**d**, **e** and **f**) were significantly changed between *Nampt*^−/−^ and *Nampt*^+/+^ (*P*-value <0.05)

To comprehensively investigate the effect of the *Nampt* gene knockout on the mouse liver transcriptome profile, we used RNA-seq to determine and compare hepatic gene expression changes between *Nampt*^−/−^ and wild-type *Nampt*^+/+^ mice. A total 4638 genes with significantly differential expression (*P*-value <0.05) in *Nampt*^−/−^ mice were identified, of which 2434 genes were upregulated and 2204 genes were downregulated (Supplementary Table 2). A total 2443 genes (52.6%) had >2-fold change (Figure 4a). Most of the genes (97.5%) were protein-coding genes, 34 genes were lincRNAs, 19 were antisense RNAs, and 232 genes coded for transcription factors. GO biological process and KEGG pathway enrichment for the 4638 genes were calculated (Supplementary Table 3 and Supplementary Table 4). The results showed that 476 biological processes and 57 significant KEGG pathways were enriched (*P*-value <0.05). GO biological process showed that 1050 genes were involved in 106 metabolic processes and 272 genes were involved in 43 biosynthetic processes. In 91 metabolic processes and 40 biosynthetic processes, there were more downregulated genes than upregulated genes (Supplementary Table 5). The examined KEGG pathways include metabolism of xenobiotics by cytochrome P450, drug metabolism, fatty acid metabolism, glutathione metabolism, nitrogen metabolism, and the citrate cycle, glycolysis/gluconeogenesis, and nicotinate and nicotinamide metabolism (Supplementary Table 4), which are pertinent to liver and Nampt functions. These results suggest that most metabolic and biosynthetic processes have been slowed down in *Nampt*^−/−^ mice.

As adult *Nampt*^−/−^ mice showed the similar intestinal villus atrophy and damage (Figure 2f) as adult replication protein A subunit3 (Rpa3, another proven essential gene) knockout mice, we compared expression levels of the liver *Rpa3* gene and three other genes(*Trp53, Atr* and *Mapk7*), whose conditional homozygous knockouts caused the death,^5, 12, 13^ between *Nampt*^−/−^ *versus Nampt*^+/+^ mice, to determine whether the *Nampt*^−/−^ caused lethality is due to downregulation of these genes. Only the *Rpa3* gene expression was significantly downregulated in *Nampt*^−/−^ mice *versus Nampt*^+/+^ mice (2.40±0.75 *versus* 5.19±0.87, Supplementary Figure 4).

As intestinal swelling (Figure 2d), villus atrophy, and damage (Figure 2f) occurred in *Nampt*^−/−^ mice, we surveyed significantly differentially expressed genes in 13-ion-associated transport processes of *Nampt*^−/−^ mouse liver tissues, which may provide information about the gastrointestinal absorption or transport of ions and nutrients from the food supply. As presented in Figure 4b, 8 out of 13-ion-associated transport processes were downregulated. Furthermore, 47.3% of genes in the whole electron transport chain (53 genes in total) were downregulated, indicating that the normal function of the electron transport chain has been compromised (Supplementary Table 6). In mitochondrial ATP biosynthesis coupled electron transport, more genes were inhibited. The same trend was true of three other ATP involved processes (Figure 4c). Of note is that the expression of 16 enzymes in TCA were downregulated, except *Pcx*, which catalyzes the carboxylation of pyruvate to oxaloacetate involved in gluconeogenesis (Figure 4d).

We further focused on the analysis of those genes involved in carbohydrate and lipid metabolism (Supplementary Table 7). Most genes in glycolysis were downregulated, but most genes in gluconeogenesis were upregulated (Figure 4e). Expressions of 29 of 32 enzymes involved in fatty acid metabolism were downregulated (Figure 4f). These findings suggest that the *Nampt* gene knockout may result in a decrease of glucose and fatty acid metabolism and an augmentation of glucose synthesis by gluconeogenesis in mouse livers. We also noticed that the expression of the lipase-encoding genes (*Lipc, Lipg*), 2, 4-dienoyl CoA reductase genes (*Decr1, Decr2*), and the enoyl-CoA isomerase (*Eci1*) were all downregulated in *Nampt*^−/−^ mice compared with the *Nampt*^+/+^ control mice. This may explain the increased accumulation of serum high polyunsaturated fatty acid containing TGs in *Nampt*^−/−^ mice compared with the *Nampt*^+/+^ control mice (Figure 3c). To investigate whether the reduced circulating phosphatidylcholine (PC) levels in *Nampt*^−/−^ mice (Figure 3d) might be explained by changes in transport, we examined the expression profiles for genes encoding PC transporters in our RNA-seq data (Supplementary Table 8). The expressions of several PC transporters, including *Abca3, Abcb4, Pcyt1a*, and *Stard7*, were decreased significantly in *Nampt*^−/−^ mice compared with the *Nampt*^+/+^ mice.

### Effect of *Nampt* gene knockout on NAD metabolism and NAD-involved gene expression

**Figure 5:**
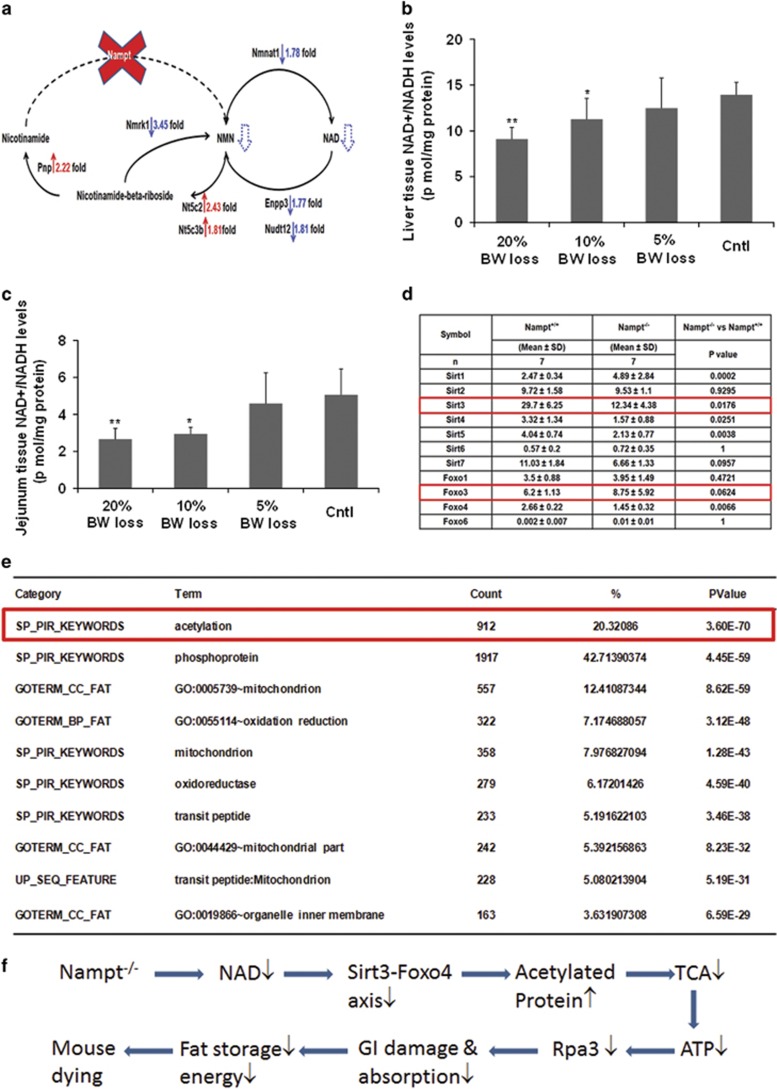
NAD metabolism and NAD-involved gene expression. (**a**) NAD synthesis from *Nampt* catalyzed salvage pathway and its metabolism in *Nampt*^−/−^ mice. Solid red arrows indicate gene upregulation while blue arrows indicate gene downregulation. Blue dotted arrows indicate reduced metabolite production. (**b**) Mouse liver NAD levels. Mouse liver tissues (30 mg) were homogenized and the supernatants were taken for NAD^+^/NADH assays as described in the 'Materials and Methods' section using a fluorimetric NAD^+^/NADH assay kit; 20, 10, and 5% BW loss groups *versus* tamoxifen-treated control (cntl) group, respectively (Bar=mean±S.D., each group *n*=5; *P<0.05, **P<0.01). BW, body weight. (**c**) Mouse jejunum tissue NAD levels. Mouse jejunum tissues (30 mg) were homogenized and the supernatants were taken for NAD+/NADH assays as described in the 'Materials and Methods' section using a fluorimetric NAD+/NADH assay kit; 20, 10, and 5% BW loss groups *versus* Tamoxifen-treated control (cntl) group, respectively (Bar=mean±S.D., each group *n*=5; *P<0.05, **P<0.01). BW, body weight. (**d**) Liver Sirt and Foxo gene expression in both *Nampt*^−/−^ *versus Nampt*^+/+^ mice. RNA-seq was carried out as described in the 'Materials and Methods' section. Gene expression levels are expressed as mean±S.D. of FPKM in each group. (**e**) Top 10 function enrichment for 4638 significantly changed genes in *Nampt*^−/−^ *versus Nampt*^+/+^ mice. (**f**) Schema of possible dying causes in *Nampt*^−/−^ mice. The explanation is provided in the 'Discussion' section

*Nampt* is a key enzyme in the cellular NAD synthesis of a mammalian salvage pathway from nicotinamide. However, the global effect and full repertoire of gene pools affected by the *Nampt* gene knockout are not fully characterized. The adult *Nampt*^−/−^ mice have afforded us the opportunity to realize this goal. Figure 5a displays that in *Nampt*^−/−^ mice, expression of nicotinamide nucleotide adenylyltransferase 1(Nmnat1), which catalyzes the formation of NAD^+^ from nicotinamide mononucleotide (NMN), was decreased significantly. Ectonucleotide pyrophosphatase/phosphodiesterase 3(Enpp3), which catalyzes the hydrolysis of pyrophosphate bonds in NAD^+^ to regenerate NMN, Nudix (nucleoside diphosphate linked moiety X)-type motif 12 (Nudt12), which cleaves NAD^+^ to AMP and NMN, and Nicotinamide riboside kinase 1 (Nmrk1), which catalyzes the nicotinamide-*β*-riboside to form NMN were all downregulated. Expression of three enzyme genes, Nt5c2: 5′-nucleotidase, cytosolic II (*Nt5c2*), 5′-nucleotidase, cytosolic IIIB (*Nt5c3b*), and Purine-nucleoside phosphorylase (*Pnp*), were all upregulated. Activation of these genes contributes to the generation of nicotinamide from NMN. All these interactions led to the decreased synthesis of NMN and hence NAD. Indeed, we found that NAD levels in liver tissue and jejunum tissue were decreased significantly in *Nampt*^−/−^ mice *versus Nampt*^+/+^ mice and paralleled to their body weight loss. In liver tissues, NAD levels are significantly lower in 20 and 15% body weight loss groups than the control group (9.10±1.27 and 11.27± 2.27 *versus* 13.94±1.35 pmole/mg tissue protein; Figure 5b). In jejunum tissue, NAD levels are significantly lower in 20 and 15% body weight loss groups than the control group (2.66±0.59 and 2.93±0.38 *versus* 5.06±1.40 pmole/mg tissue protein). In both tissues, NAD levels are decreased in 5% body weight loss group too.

NAD is a critical cofactor for sirtuin protein deacetylase. We examined the *Nampt* gene knockout on the expression of seven sirtuin genes. RNA-seq revealed that expression of Sirt3, 4, and 5 in mitochondria were decreased significantly while expression of Sirt1 in nuclei was increased significantly in *Nampt*^−/−^ mice *versus Nampt*^+/+^ mice (Figure 5d). Sirtuin protein controls mitochondrial function through the deacetylation of targets that include the FOXO protein family. We then examined the effect of sirtuin downregulation on the expression status of the liver-expressed FOXO protein family. Liver Foxo4 gene expression was inhibited significantly in the *Nampt*^−/−^ mice (Figure 5d). Sirtuin–Foxo signaling may control the expression of a number of genes. Figure 5e shows that acetylated proteins are one of the two top affected protein families, accounting for 20% of 4638 significantly differently expressed genes in *Nampt*^−/−^ *versus Nampt*^+/+^ mice.

### Co-expressed genes of human *NAMPT* gene in human prenatal and pediatric livers

**Figure 6:**
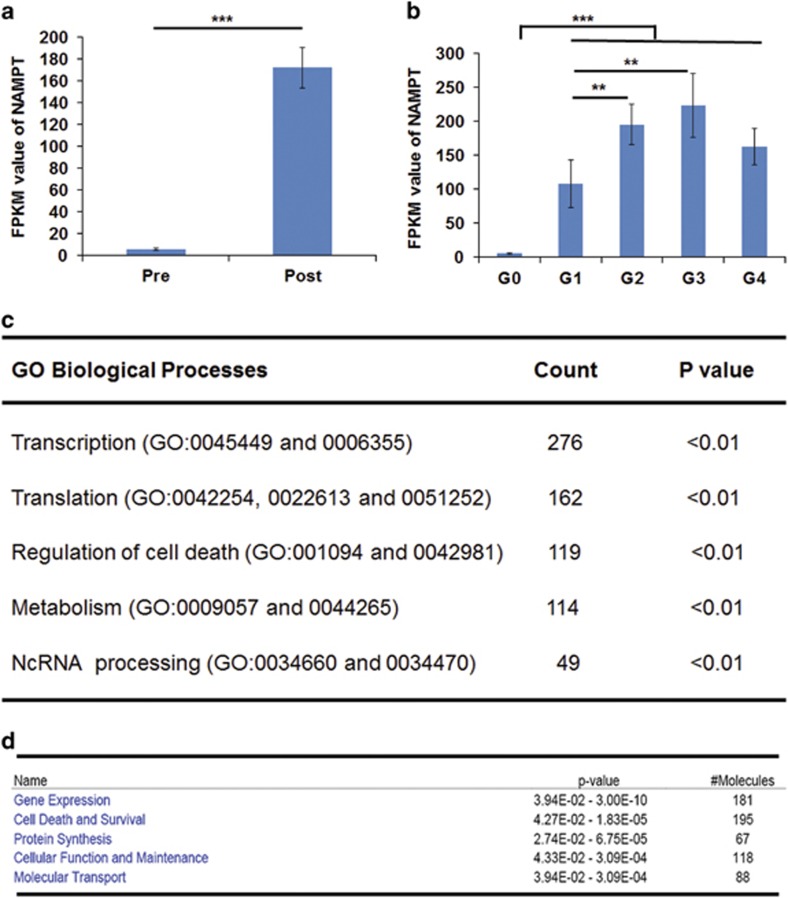
Co-expressed genes and pathway analysis of human *Nampt* gene in human pediatric liver samples (**a**) *Nampt* expression levels in prenatal and postnatal pediatric liver samples. RNA-seq analysis of these samples were carried out as described in the 'Materials and Methods'. *Nampt* expression levels are presented as FPKM. They were derived from 10 prenatal samples and 52 postnatal samples with 0–17 ages. (**b**) *Nampt* expression levels in prenatal (G0) and four different groups (G1–4) of postnatal pediatric liver samples. *Nampt* was expressed with 5.69±1.34 FPKM in G0 group. *Nampt* expression in G1 to G4 were 108.26±35.08 (G1, 0–1 year old, *n*=14), 195.06±29.80 (G2, 1–5 years old, *n*=14), 223.11±46.85 (G3, 6–11 years old, *n*=13), and 163.25±26.65 (G4, 12–17 years old, *n*=11). **P<0.01, ***P<0.001. (**c**) Top five GO biological process enrichment of co-expressed genes of human *Nampt* gene in human pediatric liver samples. (**d**) Top five molecular and cellular functions of co-expressed genes of human *Nampt* gene in human pediatric liver samples

To relate what we learned about potential functions of the *Nampt* gene in mouse liver to *NAMPT*'s role in human liver, we examined the expression patterns of the *NAMPT* gene and its co-expressed genes in human prenatal and pediatric liver from an existing RNA-seq data set. The goal of this ‘guilt-by-association' coexpression relationship analysis was to provide insights into potential functional roles of *NAMPT* and associated genes. After birth, expression of the *NAMPT* gene was upregulated significantly (171.98±18.47 FPKM postnatally, *n*=52 *versus* 5.69±1.34 FPKM prenatally, *n*=10, *P*<0.01; Figure 6a). Expression of *NAMPT* genes positively correlated with the children's development and growth until puberty (Figure 6b). A total 1064 genes displayed the same expression pattern as the *NAMPT* gene (Supplementary Table 9). GO biological process enrichment of these co-expressed genes found that they are involved in several important processes: transcription, translation, regulation of cell death, metabolism, and noncoding RNA processing (Figure 6c). The top five involved molecular and cellular functions of these co-expressed genes include gene expression, cell death and survival, protein synthesis, cellular functions and maintenance, and molecular transport (Figure 6d). These results suggest that NAMPT may have similar roles in these processes and functional categories.

## Discussion

Our study represents the first scientific report, with concrete experimental evidence in a C57BL/6J mouse model, that *Nampt* is an essential gene *in vivo*. This claim is supported by the findings that the *Nampt* gene homozygous knockout (*Nampt*^−/−^) mice were embryonically lethal and *Nampt*^−/−^ adult mice died within 5–10 days with 25.24±2.22% body weight loss. Although several groups – including ours – previously noted that *Nampt* gene homozygous knockout mice were embryonic lethal, none of them reported physical evidence.^3, 14, 15, 16, 17^ Here, we have presented the morphological and genotyping evidence of mouse embryos at both 8.5 and 11.5 d.p.c. (Figures 1d and e), clearly demonstrating that *Nampt*^−/−^ mouse embryos had significantly smaller sizes and abnormal gross structures, and died before organogenesis. This ‘gold standard' of evidence authenticates that the *Nampt* gene is an essential gene in living organisms.

In adult *Nampt*^−/−^ mice, we found seven phenotypes: death at 5–10 days after the induction >20% body weight loss (Figure 2); decreased cell NAD levels of both liver and intestinal tissues (Figures 5b and c); intestinal swelling (Figure 2d); atrophic intestinal villi (Figure 2f); visceral fat depletion (Figure 2d); higher serum TG and lower serum PC levels (Figure 3). To unravel the molecular underpinnings of these processes, we utilized RNA-seq^18^ to profile mouse liver transcriptomes to gain transcriptional insights into the essential role of *Nampt* in living organisms. Several important themes have emerged. First, 4638 differentially expressed genes in *Nampt*^−/−^ mouse livers (Figure 4a and Supplementary Table 2) affect 476 biological processes and 57 significant KEGG pathways, corroborating *Nampt*'s pleiotropic functions. Second, our study found that liver tissue and jejunum tissue NAD levels were decreased significantly in *Nampt*^−/−^ mice *versus* *Nampt*^+/+^ mice (Figures 5b and c), confirming Nampt's key role in cell NAD synthesis.^2, 16, 19^ RNA-seq revealed that in *Nampt*^−/−^ mice, key enzymes in two other salvage pathways and the *de novo* synthesis pathway of NAD synthesis were significantly decreased. It would be of interest to determine whether NAMPT directly or indirectly inhibits expression of these genes. Third, RNA-seq analysis of mouse liver transcriptomes in *Nampt*^−/−^ mice *versus Nampt*^+/+^ mice have provided a number of novel insights into the essential role of the *Nampt* gene. In *Nampt*^−/−^ mice, genes encoding acetylated proteins and genes in gluconeogenesis were upregulated while genes in glycolysis, lipase and tricarboxylic acid cycle were downregulated (Figure 4d). TCA shutdown would decrease ATP synthesis. Without enough ATP, those ATP-dependent transporters – such as those in intestine involved in nutrient absorption or transport – would cease to function. RNA-seq analysis indicated that expression of most ion transporter genes were inhibited significantly in *Nampt*^−/−^ mice (Figure 4b). In addition, ATP is needed to maintain normal tissue structural integrity. In *Nampt*^−/−^ mice, the short supply of ATP may be responsible for disrupted intestinal architecture and villus atrophy (Figure 2f). This may also be due to the high rate of cell turnover in this tissue, which makes it more susceptible to energy deficiency. This could explain why, in clinical trials of cancer treatment, oral administration of FK866 or CHS828,^20, 21^ which inhibits NAMPT enzymatic activity, caused unwanted side effects dominated by gastrointestinal symptoms. It suggests that alternative routes of drug administration may avoid or minimize gastrointestinal side effects, and achieve better therapeutic efficacy. Of interest is that we found that *Rpa3* gene expression was decreased by more than 50% in *Nampt*^−/−^ mice (Supplementary Figure 4). The phenotype of disrupted intestinal architecture and villus atrophy in *Nampt*^−/−^ mice looks similar to those in *Rpa3*^−/−^ mice.^5^ It remains to be elucidated whether this effect is due to the direct regulation of the *Nampt* gene knockout on *Rpa3* gene expression.

NAD is a critical cofactor for sirtuin protein deacetylase. In *Nampt*^−/−^ mice, expression of mitochondrial Sirt3, Sirt4, and Sirt 5, especially Sirt3, were all diminished significantly while nuclear Sirt1 was augmented (Figure 5f), suggesting that Sirt3, instead of Sirt1, may have a key role in life among seven sirtuin proteins. This is in line with previous evidence by Yang *et al*.^22^ Sirtuins can regulate transcription by deacetylating transcription factors such as FOXO family. Among several common *FOXO* genes, expression of FOXO4 was inhibited significantly. The dysregulated Sirt3–Foxo4 axis may set up a chain of reaction to engender the dysregulation of a number of genes in *Nampt*^−/−^ mice, becoming a death axis in *Nampt*^−/−^ mice. Based on the transcriptional insights, we sum up a tentative schema in Figure 5f to explain *Nampt*^−/−^-induced death process. Briefly, the homozygous *Nampt* gene knockout causes a decrease in cell NAD synthesis, which leads to the dysregulation of NAD-dependent genes such as those encoding acetylated proteins including Sirt3–Foxo4 axis and especially the suppression of eight enzymes in TCA cycle. The resultant decreased ATP output may adversely affect other genes including *Rpa3*, ion and nutrient transporters, as well as disrupt normal intestinal tissue architecture, which hinders normal nutrient absorption and transport into the visceral organs and forces the mobilization and exhaustion of fat storage and all energy sources, culminating in mouse death.

It should be pointed out that *Nampt*'s functional roles may not solely depend on its enzymatic activity on NAD synthesis.^23, 24, 25^ In *Nampt*^−/−^ mice, liver and intestinal NAD levels were only 20–30% lower than those in surviving adult *Nampt*^+/+^ mice. Thus, it is warranted to explore whether the disruption of non-enzymatic functions of *Nampt* also has a critical role in the dying phenotype of adult *Nampt*^−/−^ mice.

Although most data in this study were derived from mice, transcriptional insights from the mouse liver transcriptome are largely confirmed by data derived from co-expressed genes of the *NAMPT* gene in human prenatal and pediatric liver samples (Figures 6a–d and Supplementary Table 9). These results strongly indicate that our findings are applicable to human studies. *NAMPT* has been implicated in the pathogenesis or therapeutic targets of a number of human diseases or conditions,^4^ such as acute respiratory distress syndrome,^26^ aging,^27^ cancer,^28^ coronary artery disease,^29^ diabetes, nonalcoholic fatty liver disease and obesity,^30^ rheumatoid arthritis, and sepsis.^31^ Recently, a small chemical molecule, P7C3 and its derivatives, has been found to exert neuroprotective activity in animal models of Parkinson's disease, amyotrophic lateral sclerosis, and concussive injury.^32^ This compound was identified as an allosteric activator of NAMPT.^33^ Our study contributes global metabolic and transcriptional insights into Nampt's essential roles and also provides potential leads to *Nampt*'s pleiotropic physiological functions and pathological roles applicable to disease in humans.

## Materials and Methods

### Generation of *Nampt*^+/−^ mice and their timed pregnancy

A mouse (129/Ola) embryonic stem (ES) cell line (RRR084) harboring characterized insertional mutation in the intron-7 of mouse *Nampt* gene via gene-trap vector inactivation was obtained from BayGenomics (http://baygenomics.ucsf.edu), a consortium of research groups in the San Francisco Bay Area funded as part of the NHLBI's Programs for Genomic Applications.^34^ The verified RRR084 clone was injected into C57BL/6N (NCI) blastocysts to generate chimeras in the Johns Hopkins University School of Medicine (Baltimore, MD, USA) transgenic facility. The generated chimeric *Nampt*^+/−^ mice were genotyped by PCR of their tail DNAs. The chimeric *Nampt*^+/−^ mice were backcrossed with C57BL/6J for 10 generations before their usage. All mice were fed an AIN-76 A diet and water *ad libitum*. They were housed under controlled conditions (25±2 °C; 12 h light–dark periods). All mouse experiments were conducted in accordance with the NIH guidelines and were approved by the University of Missouri Kansas City Animal Care and Use Committee.

The early stages of *Nampt* gene mutant mouse embryos were obtained by timed pregnancy after breeding between *Nampt*^+/^^−^ male and *Nampt*^+/−^ female mice. Visual observation of a vaginal plug in female *Nampt*^+/−^ mice after the mating was counted 0.5 days post coitum (d.p.c.). Mouse embryos at 8.5 or 11.5 d.p.c. were harvested and fixed in 4% paraformaldehyde for 24 h. The genotyping of mouse embryos was done by PCR amplification of yolk sac DNA. Mouse embryos at 8.5 d.p.c. were also subjected to β-Gal staining.

### Generation of *Nampt* gene exon 2 floxed (*Nampt*^F/F^) and *Nampt*^−/−^ mice

A *Nampt* gene target vector prepared in our lab, in which the exon 2 of *Nampt* gene was floxed, was electroporated into a B6-White Murine ES Cell Line (cat. no. SCR011, EMD Millipore, Billerica, MA, USA) and injected into C57BL/6J mouse blastocysts in The Transgenic Animal Core, University of Missouri, Columbia, USA. The generated chimeric *Nampt*^F/F^ mice were verified by PCR analyses of their tail DNAs.

Adult *Nampt*^−/−^ mice were generated by mating *Nampt*^F/F^ mice with B6.Cg-Tg(CAG-cre/Esr1*)5Amc/J(Stock No:004682, Jax Lab, Bar Harbor, ME, USA) followed by administering tamoxifen induction.

### Histological examination of mouse intestinal tissues

Formalin-fixed mouse intestinal tissue samples were embedded in paraffin, and 5-micron-thick sections were cut. Replicate sections were stained with hematoxylin and eosin (H & E) according to standard methods.

### Mass Spec analysis of mouse serum lipid levels

The serum samples were collected and stored at −80 °C until analyzed. The samples were extracted and subjected to the ultra-high performance liquid chromatography – high resolution mass spectrometry UHPLC-HRMS procedure as outlined by Oliver Fiehn's group^35^ and (http://www.metabolomicsworkbench.org/protocols/getprotocol.php?file_id=163). The analysis was conducted on a Waters Acquity UPLC coupled to a Waters Xevo G2-qTOF (quadrupole time-of-flight) mass spectrometer. The samples were analyzed in positive electrospray mode, and the data were acquired using the MSe acquisition format with a scan rate of 2 Hz. High-energy MSe scans were ramped from 15 eV to 30 eV. A leucine enkaphlin (2 *μ*g/ml) solution was used as lockspray solution. Data processing, including chromatographic alignment and peak picking occurred in Progenesis QI 2.0. Potential markers were identified by matching the molecular ion (5 p.p.m. error) and fragment ions (50 p.p.m. error) to the lipidblast database using the database search feature within progenesis QI. Further processing occurred by exporting the data to EZinfo 3.0.

### Mouse liver RNA isolation

Total mouse liver RNA was isolated from either the wild-type mice or *Nampt*^−/−^ mice using the MirVana kit (cat. no. AM1560, ThermoFisher Scientific, Grand Island, NY, USA) according to the manufacturer's instructions.

### RNA sequencing of mouse liver transcriptomes

RNA-seq of mouse liver RNAs was carried out in the Core of Genetic Research, Children's Mercy, Kansas City, KS, USA, according to the previously published protocol^36^ except that sequencing library from 1 *μ*g total mouse liver RNA of each sample was prepared using the Illumina TruSeq Stranded Total RNA Sample Prep Kit (cat. No. RS-122-2201) and 2 × 101 paired-end sequencing of high output run mode was performed using the HiSeq 1500 instrument. Each sample was sequenced to an average depth of ~63 million reads with >80% of bases above Q30. The resulting base calling (.bcl) files were converted to FASTQ files using Illumina's bcl2fastq v2.17.1.14 software. Mapping of RNA-seq reads and transcript assembly and abundance estimation were conducted using Tuxedo Suite pipeline (TopHat v1.3.0/Bowtie v0.12.7/Cufflinks v1.0.3) and reported in Fragments Per Kilobase of exon per Million fragments mapped (FPKM).

### Functional analysis of differentially expressed gene lists using Ingenuity Pathway Analysis

The pathway analysis of differentially expressed gene lists in *Nampt*^−/−^ mice were analyzed using DAVID (https://david.ncifcrf.gov/)^37^ and Ingenuity Pathway Analysis (IPA, Ingenuity Systems, Inc., Redwood City, CA, USA) as we described before.^38, 39^ Briefly, genes with a significant expression change (*P*<0.05) in mouse liver expression in *Nampt*^−/−^ mice *versus* wild-type mice were loaded into IPA and they were associated with various pathways in Ingenuity's Knowledge Base for the analysis. The significance of the association between the data set and any pathway was measured in two ways: (i) the ratio of the number of molecules from the data set that map to the pathway divided by the total number of molecules that map to the pathway, and (ii) Fisher's exact test was used to calculate a *P*-value determining the probability that the association between the genes in the data set and the pathway in IPA is explained by chance alone. Transcription factor prediction database (DBD)^40^ and Database of Essential Genes (DEG)^41^ were used to annotate mouse transcript factors and essential genes for differentially expressed gene list.

### Human pediatric liver samples and RNA-seq analysis

Forty-eight, anonymized pediatric liver samples, ages 0 days to 17 years, were received from the following sources: the NICHD Brain and Tissue Bank at the University of Maryland (funded by NIH contract HHSN275200900011C, Ref. No. #N01-HD-9-0011); the Liver Tissue Cell Distribution System (funded by NIH Contract #N01-DK-7-0004/HHSN267200700004C); and six samples were received from XenoTech, LLC (Lenexa, KS, USA), to whom we are grateful for their generous gift of tissue samples. Thirty-four prenatal human liver samples (57–196 days, post-conception) were received from an additional NICHD-funded tissue retrieval center, the Central Laboratory for Human Embryology (University of Washington, Seattle, WA, USA). All tissues were flash frozen and maintained at −80 °C before isolation of DNA and RNA. All the samples were from anonymous, deceased donors, and the use of these tissues was declared as nonhuman subjects research by the University of Missouri-Kansas City Pediatric Health Sciences Review Board.

RNA-seq analyses of human pediatric liver samples were carried out as described above for mouse liver transcriptome analyses.

### Measurement of mouse liver and jejunum NAD+/NADH levels

Mouse liver and jejunum tissues (10–30 mg) were homogenized and the supernatants were applied for NAD+/NADH assays using a Fluorimetric NAD+/NADH assay kit (Cat. No. 15263; AAT Bioquest. Inc. Sunnyvale, CA, USA).

### Statistical analyses

Statistical analyses were performed using the Sigma Stat (ver 13.0, Systat Software, Inc., San Jose, CA, USA). Results are expressed as means±S.D. of more than three samples for each group from at least two independent experiments. Two group comparisons were done by unpaired *t-*test. Three or more group comparisons were carried out using ANOVA followed by a Holm–Sidak test, *P*<0.05 was considered statistically significant.
