# Supplementary material for: Metabolic and molecular insights into an essential role of nicotinamide phosphoribosyltransferase
Source: Cell Death Dis. 2017 Mar 23;8(3):e2705–. doi: 10.1038/cddis.2017.132 (PMC5386535; doi:10.1038/cddis.2017.132)
Supplement: Supplementary Information [file cddis2017132x1.docx]

**Metabolic and molecular insights into an essential role of nicotinamide phosphoribosyltransferase***

Li Q. Zhang^1^, Leon Van Haandel^2^, Min Xiong^1^, Peixin Huang^1^, Daniel P. Heruth^1^, Charlie Bi^2^, Roger Gaedigk^2^, Xun Jiang^1,4^, Ding-You Li^3^, Gerald Wyckoff^5^, Dmitry N. Grigoryev^6^, Li Gao^7^, Linheng Li^8^, Min Wu^9^, J. Steven Leeder^2^*,* Shui Qing Ye^1,10, #^

From Division of Experimental and Translational Genetics^1^, Division of Clinical Pharmacology and Therapeutic Innovation^2^, Division of Gastroenterology^3^, Department of Pediatrics, The Children's Mercy Hospital, Kansas City, MO, USA; Department of Pediatrics^4^, Tangdu Hospital, Fourth Military Medical University, Xian, China; Division of Molecular Biology and Biochemistry^5^, University of Missouri School of Biological Sciences, Kansas City, MO, USA; Laboratory of Genitourinary Cancer Pathogenesis^6^, National Cancer Institute, Bethesda, MD, USA; Division of Allergy & Clinical Immunology^7^, Johns Hopkins University School of Medicine, Baltimore, MD, USA; Stowers Institute for Medical Research^8^, Kansas City, MO, USA; Department of Biomedical Sciences^9^, University of North Dakota, Grand Forks, North Dakota, USA; and Department of Biomedical and Health Informatics^10^ , University of Missouri Kansas City School of Medicine, Kansas City, MO, USA

*Running title: **Essential role of NAMPT**

^#^ To whom correspondence should be addressed:

Shui Qing Ye, MD, PhD

Division of Experimental and Translational genetics

Department of Pediatrics

The Children’s Mercy Hospital

University of Missouri Kansas City School of Medicine

Kansas City, MO, USA

Fax: (816) 983-6501

E-mail: [sqye@cmh.edu](mailto:sqye@cmh.edu)

**Supplement files**

**Figure legends**

**Fig. S1. Partial mouse Nampt gene sequence (Exon 7, Intron 7, Exon 8) and gene trap vector (pGT0lxf) insertion Site.** PCR amplified mouse Name gene fragment was sequenced by Sanger sequencing. Both exon 7 and exon 8 sequences are marked in blue. The intron 7 sequences are displayed in black. Genotyping primers are marked in red. The red arrow head indicates the gene trap vector (pGT0lxf) insertion Site (-499 bp from the exon 8).

**Fig. S2. Generation of Nampt ^F/+^ chimeric mice.** Nampt ^F/+^ mice were derived from a B6-White™ Murine ES Cell Line into which a Nampt gene targeting vector was electroporated as described in the Methods. **A. A representative chimeric Nampt ^F/+^ mouse.** A B6-albino mouse and a C57BL/6J mouse are also displayed to show the contrast colors. **B. Genotype Nampt ^+/+^ and Nampt ^-/-^ mice.** A representative genotyping gel image of a Nampt ^+/+^ mouse (133) and a Nampt ^-/-^ mouse (134) in two different tissues (heart, H and kidney, K) is shown.

**Fig. S3. Heat map of mouse serum lipid profiles as detected by Mass Spectrometry**. Serum lipid profiles from four different groups of mice plus one pooled samples were measured by Mass Spectrometry as described in Methods. A heat map of 141 lipids is presented. Each column represents a sample. Each role represents a lipid. Each group is marked in different color on the top. Red color indicates higher amount and blue color lower amount of lipid. N=3/ each group except the pooled group (n=4).

**Fig. S4. Liver Rpa3 gene expression levels between Nampt ^-/-^ mice vs Nampt^+/+^ mice.** RNA-seq of mouse liver RNAs was carried out as described in the Method. Statistical comparison of several liver gene expressions between Nampt ^-/-^ mice vs Nampt^+/+^ mice was done by Student T-test. The mean ± SD values for Trp53, Atr, Rpa3, and Mapk7 are 5.28±2.64 vs 4.59±0.79, 2.31±0.45 vs 1.78±0.36, 2.40±0.75 vs 5.19±0.87, and 1.89±0.61 vs 0.70±0.20, respectively. Trp53: Tumor Protein P53, Atr: ATR Serine/Threonine Kinase, Rpa3: Replication Factor A Protein 3, Mapk7: Mitogen-Activated Protein Kinase 7. *, p<0.01, n=7 for each group.

**Supplement Table (Table S) legends**

**Table S1. Mouse serum lipid detections by a UHPLC-qTOF MS-based serum metabolic profiling.** The serum lipid content in four experimental groups (Vehicle control, Tamoxifen control, 10% Weight Loss, 20% Weight Loss; N= 3 per group) was measured by UHPLC-qTOF. As an internal control, equal amounts of serum from each experimental mouse group were pooled into 4 control groups and analyzed simultaneously with the experimental groups.

**Table S2. 4638 genes with significant expression changes in Nampt-/- vs wild type Nampt+/+ mice.** 4638 mouse liver genes with significant expression changes in Nampt-/- vs wild type Nampt+/+ mice are presented. N=7/each group. The expression of all genes were significantly changed between Nampt-/- and Nampt+/+ mice (p value < 0.05).

**Table S3. Genes with significant expression changes (P<0.05) in Nampt-/- mice vs wild type Nampt+/+ mice in metabolic and biosynthetic processes.** The significant expression change genes (P<0.05) in Nampt-/- mice vs wild type Nampt+/+ mice in metabolic and biosynthetic processes are presented. All metabolic and biosynthetic processes derived from gene biological process ontology and were tested by Fisher exact test as a significant difference (p < 0.05).

**Table S4. Kegg pathway enrichment for 4638 significant changed genes in Nampt-/- vs wild type Nampt+/+ mice.** Kegg pathway enrichment for 4638 significant changed genes in Nampt-/- vs wild type Nampt+/+ mice is presented. Fisher exact test was used to determine the significant enrichment by p value < 0.05.

**Table S5. Statistical summary of significant expression genes in metabolic and biosynthetic processes.** Statistical summary of significant expression genes in metabolic and biosynthetic processes is presented.

**Table S6. Genes with significant expression changes (p<0.05) in ATP associated processes in Nampt-/- vs Nampt+/+ mice.** Genes with significant expression changes (p<0.05) in ATP associated processes in Nampt-/- vs Nampt+/+ mice are presented.

**Table S7. Selected genes with significant expression changes (p<0.05) in lipid metabolism in Nampt-/- vs Nampt+/+ mice.** Selected genes with significant expression changes (p<0.05) in lipid metabolism in Nampt-/- vs Nampt+/+ mice are presented. N=7/each group. The expression of all genes were significantly changed between Nampt-/- and Nampt+/+ mice (p value < 0.05).

**Table S8. Selected genes with significant expression changes (p<0.05) in PC transports in Nampt-/- vs Nampt+/+ mice.** Selected genes with significant expression changes (p<0.05) in PC transports in Nampt-/- vs Nampt+/+ mice are presented. N=7/each group. The expression of all genes were significantly changed between Nampt-/- and Nampt+/+ mice (p value < 0.05).

**Table S 9. Coexpressed genes of the NAMPT gene in human prenatal and pediatric liver tissues.** Coexpressed genes of the NAMPT gene in human prenatal and pediatric liver tissues are presented. Weighted correlation network analysis (WGCNA), an R package for weighted correlation network analysis (https://labs.genetics.ucla.edu/horvath/CoexpressionNetwork/Rpackages/WGCNA/), was utilized to identify human NAMPT coexpressed genes in 10 prenatal samples and 52 postnatal samples with 0-17 ages. All the coexpression genes were selected with Bonferroni-adjusted p-value ≤ 0.05.
