# Supplementary figures and images for: Metabolic and molecular insights into an essential role of nicotinamide phosphoribosyltransferase
Source: Cell Death Dis. 2017 Mar 23;8(3):e2705–. doi: 10.1038/cddis.2017.132 (PMC5386535; doi:10.1038/cddis.2017.132)

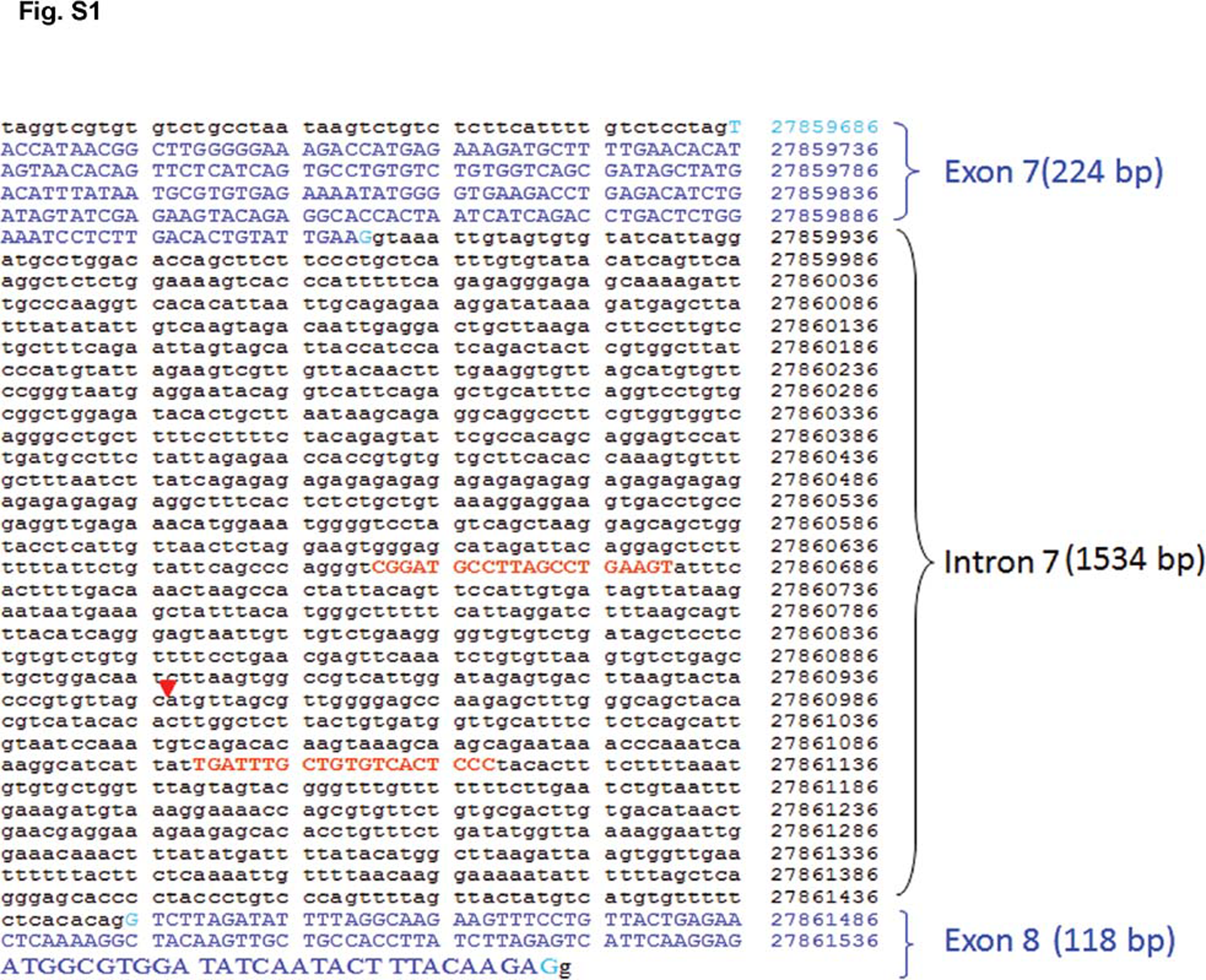

Supplement: Supplementary Figure 1 [file cddis2017132x2.tif]

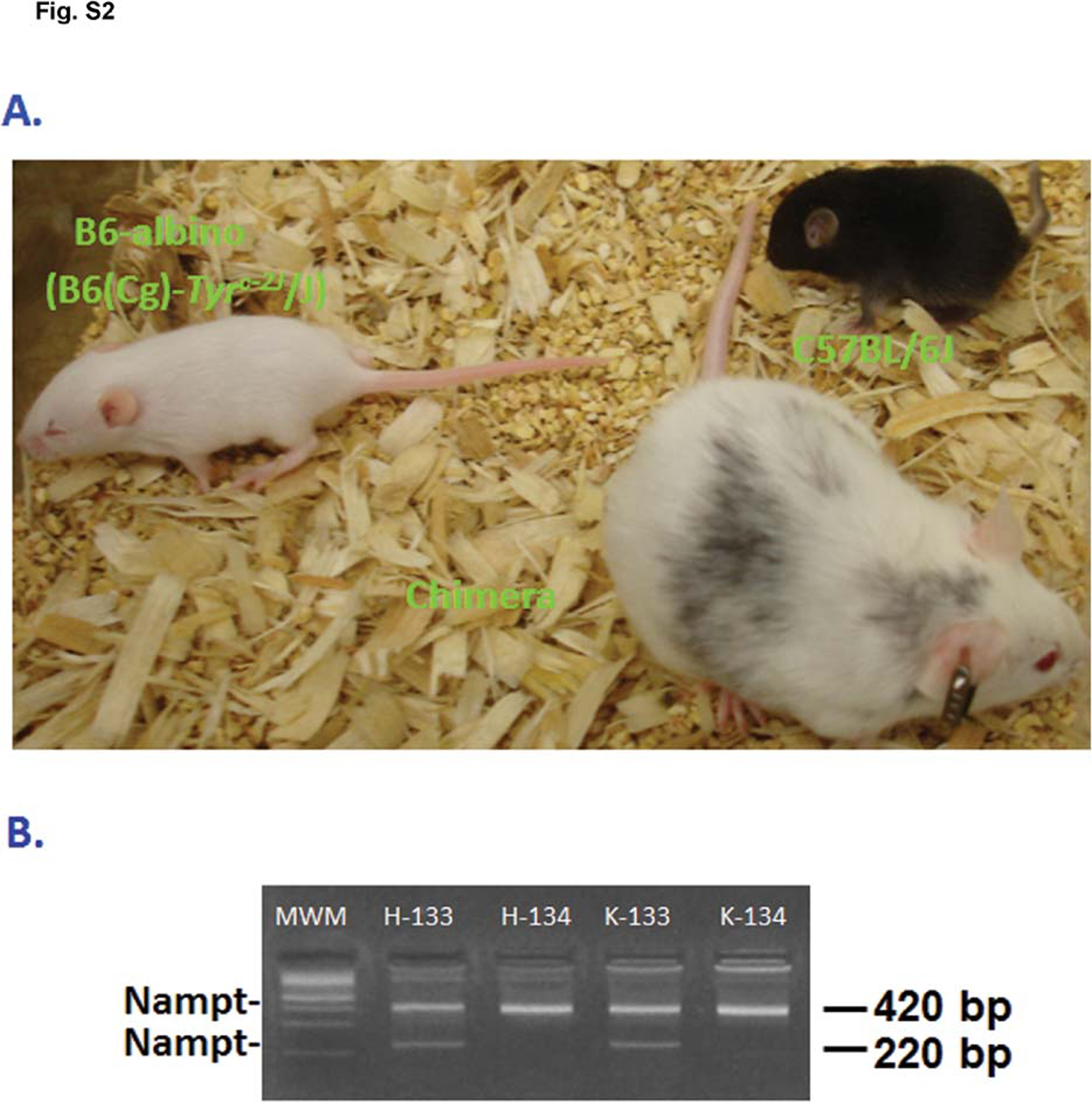

Supplement: Supplementary Figure 2 [file cddis2017132x3.tif]

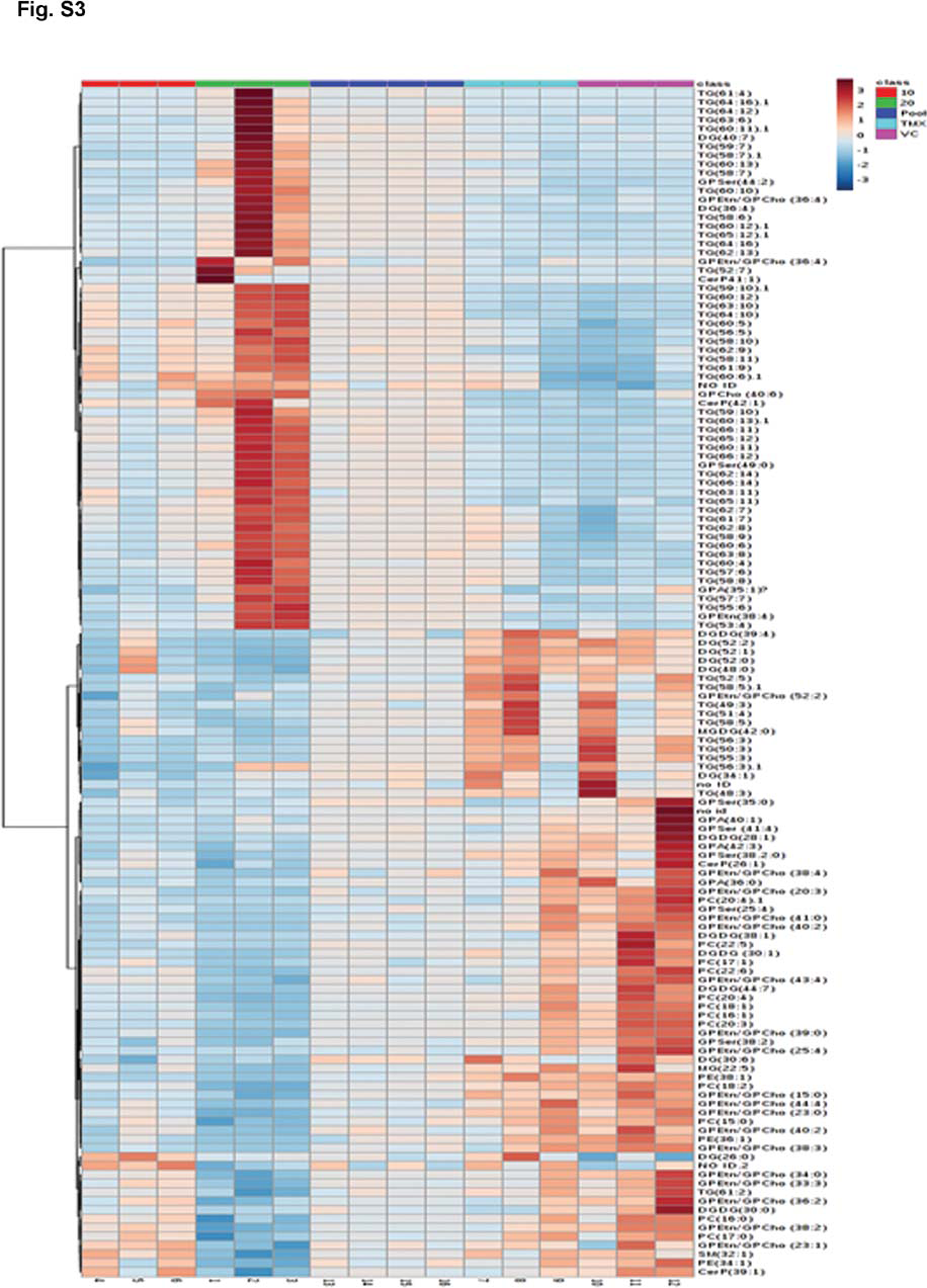

Supplement: Supplementary Figure 3 [file cddis2017132x4.tif]

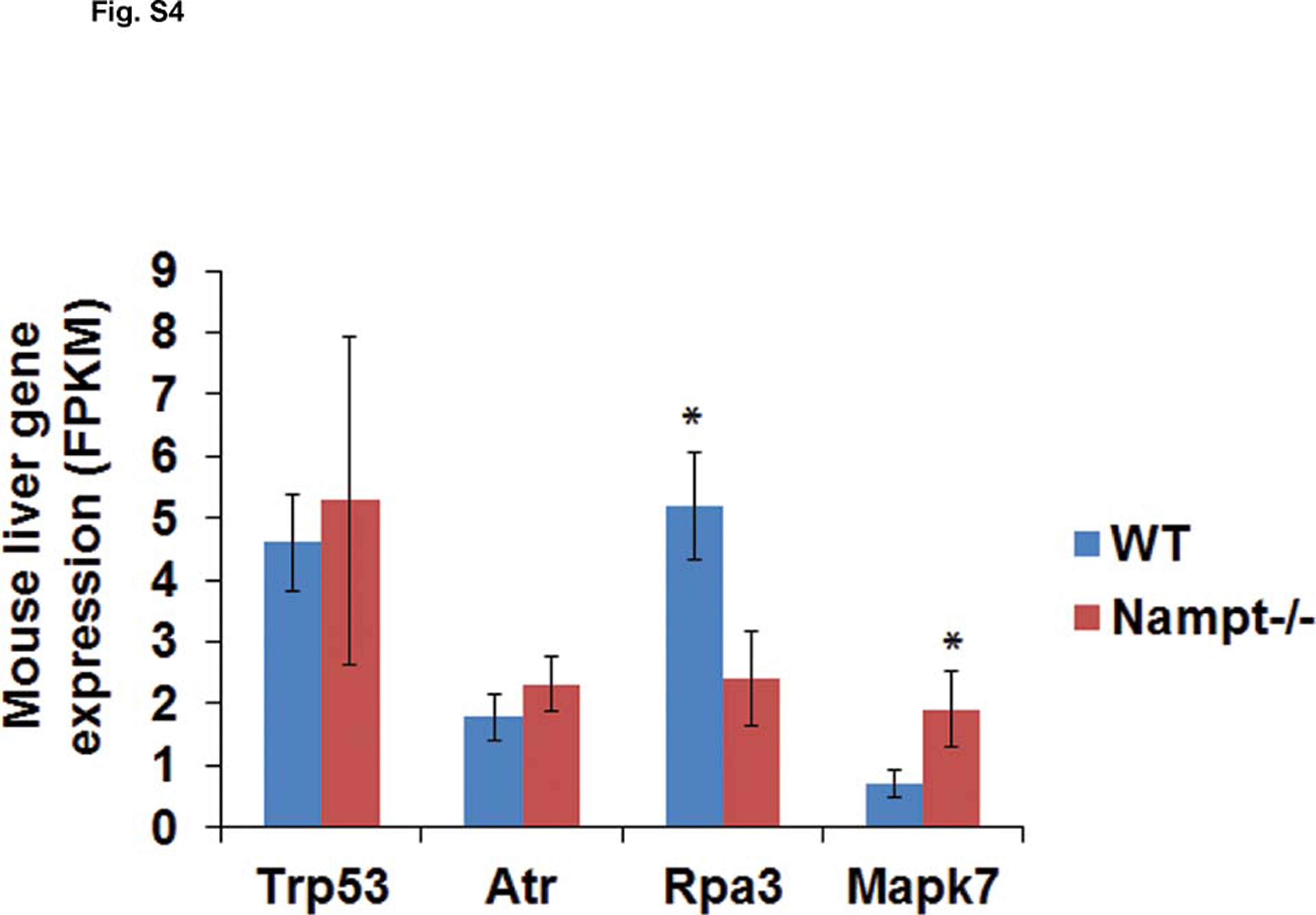

Supplement: Supplementary Figure 4 [file cddis2017132x5.tif]
